# Ceramide as a Biomarker for HFpEF in Women: Menopause, Aging, and Pregnancy

**DOI:** 10.3390/ijms262110800

**Published:** 2025-11-06

**Authors:** Ruth R. Magaye, David M. Kaye, Bing H. Wang

**Affiliations:** 1 Heart Failure Research Group, Baker Heart and Diabetes Institute, Melbourne, VIC 3004, Australia; 2 Monash Alfred Baker Centre for Cardiovascular Research, Monash University, Clayton, VIC 3800, Australia; 3 Department of Cardiology, Alfred Hospital, Melbourne, VIC 3004, Australia; 4 Biomarker Discovery Laboratory, Baker Heart and Diabetes Institute, Melbourne, VIC 3004, Australia

**Keywords:** heart failure with preserved ejection fraction, ceramides, females, hypertension, obesity, aging

## Abstract

Heart failure with preserved ejection fraction (HFpEF) currently accounts for half of the heart failure (HF) cases world-wide, affecting nearly 32 million people. HFpEF has a skewed prevalence toward females and those older than 65 years old. The pathophysiology of HFpEF is suggestive of a conglomerate of inflammatory, hypertensive, as well as metabolic dysfunction, giving rise to the syndrome. Disruptions in ceramide metabolism do occur in heart failure as well as within the HFpEF-associated risk factors, both modifiable inflammation, obesity, hypertension, diabetes, and non-modifiable-aging, and female sex. The focus of this review is to draw attention to the links between changes in female biophysiology, such as pregnancy, menopause and aging, in which ceramide is dysregulated and consequently gives rise to the same pathologies that are labeled as risk factors for HFpEF. Our objective is to highlight ceramides as potential biomarkers for prevention and initial diagnostic tools for HFpEF, especially for women later in life.

## 1. Introduction

Perturbations in lipid biosynthesis and metabolism are emerging as targets for biomarkers in chronic and often evasive diseases such as HFpEF (heart failure with preserved ejection fraction) [1,2]. The sphingolipid ceramide has gained attention in recent years for its association with increased risk for CVDs (cardiovascular diseases) including heart failure [3,4,5,6,7,8]. The mechanisms and relationships between ceramide and the heart failure phenotype HFpEF are paramount to understanding the pathophysiology of HFpEF. HFpEF accounts for nearly 50% of HF (heart failure) cases and has predictions of an annual increase in prevalence of 1% above HFrEF (HF with reduced ejection fraction) [9,10]. HFpEF risk factors include hypertension, obesity, diabetes, as well as female sex and aging. These risk factors often co-exist and confound HFpEF diagnosis and treatment. An important aspect of HFpEF is its skewed prevalence toward the female sex, accounting for 50–60% of cases [10,11,12]. This sexual dimorphic nature of HFpEF points to the obvious physiological differences between the male and female life terms, such as the impact of pre- and post-menopausal estrogen-E2 (17β-estradiol) fluctuations and obstetric factors on female cardiovascular health [13,14,15,16]. Ceramide levels are not only known to fluctuate in line with these different stages in the female life term [17,18,19], but also within the context of the other HFpEF risk factors [20,21,22,23,24]. An event that ceramide is known to impact in the female life cycle is pregnancy. Ceramides act as stress mediators during implantation, delivery, and lactation [19], and its levels are increased in pregnant versus non-pregnant women [25,26]. The chances of having CVDs, including HFpEF, increase with pregnancy-related complications such as GDM (gestational diabetes mellitus) and hypertensive disorders in women [27,28,29]. Circulating ceramide levels are increased in these pregnancy complications and have been investigated as potential biomarkers for early detection or prediction [22,30]. Age also impacts plasma ceramide levels, with higher levels observed in post-menopausal (47–78 years old) compared to pre-menopausal women [17]. Since HFpEF is prevalent in females aged ≥60 years of age, understanding how these changes in ceramide over the female life term contribute to the risk factors of HFpEF may lead to ceramides being a potential biomarker for HFpEF in females. In this review, we start with a brief overview of the complexities involved in ceramide biosynthesis that can feed into ceramide dysregulation in multifactorial diseases such as HFpEF. We also evaluate and highlight how ceramide plays a role in multiple HFpEF risk factors, such as hypertension, obesity, diabetes, and aging in females, especially those with a history of multiple pregnancies and pregnancy-associated disorders. while demonstrating this we highlight the lack of female-focused research in terms of these risk factors over the life of the female.

## 2. Brief Overview of Ceramide Biosynthesis and Biology

**Figure 1:**
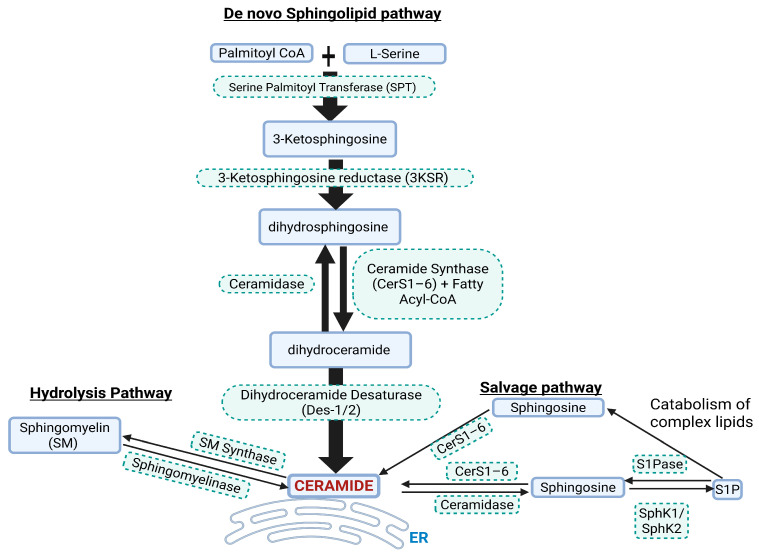
Simplified pathways for Cer synthesis. ER = endoplasmic reticulum, SphK1/SphK2 = sphingosine kinase 1 and 2, S1P = sphingosine 1 phosphate, S1Pase = sphingosine 1 phosphatase.

Ceramide is synthesized through the de novo sphingolipid biosynthesis pathway Figure 1). Ceramide synthesis takes place at the leaflet of the ER (endoplasmic reticulum), where SPT (serine palmotyl transferase) catalyzes palmitoyl-CoA with L-serine, subsequently forming 3KS (3 ketosphinganine). 3KS is reduced to dhSph (dihydrosphingosine) by 3KS reductase. CerS1–6 (ceramide synthases 1–6) then acylate dhSph with a fatty acyl-CoA to form dhCer (dihydroceramide) with defined chain lengths (C14–C26). Ceramide chain length is important in diseases, including CVDs. The insertion of a 4–5 trans-double bond in dhCer by the enzymes DES1–2 (dihydroceramide desaturase 1 and 2) results in the formation of ceramide. Ceramide is then transported from the ER to other sites such as the trans-Golgi apparatus at membrane contact sites via vesicle and non-vesicle transport systems to produce complex sphingolipids such as glucosylceramide [31]. Ceramide can also be generated from enzymatic degradation of complex sphingolipids through SM (sphingomyelin) hydrolysis or the salvage pathways [32]. The SM hydrolysis pathway involves SMase (sphingomyelinase) converting SM to ceramide and the salvage pathway involves CerS1–6 that salvage or recycle ceramide from the catabolic breakdown of the sphingolipid backbone, resulting in production of Sph (sphingosine) which is re-acylated to ceramide. Multiple enzymes are involved in the synthesis of ceramides, and targeting these in in vivo and in vitro models has shown how ceramide can play a role in diseases. For example, targeting ceramide synthesis via ablation of genes such as *Spltc2*, *degs1,* and *CerS* or pharmacological inhibition of the enzymes they code for leads to reductions in ceramide accumulation and disease progression [33]. The CerS1–6 are crucial enzymes involved in catalyzing the final step in ceramide formation by attaching fatty acids to the sphingolipid backbone. These enzymes exhibit distinct substrate specificity for fatty acyl-CoA, leading to generation of ceramides with specific acyl chain lengths [34]. CerS1 primarily leads to the generation of C18 ceramides, CerS2–3 synthesizes very long-chain (C20-C26) ceramides, CerS4 synthesizes C18-C20 ceramides, while CerS5–6 synthesizes C14 and C16 ceramides [35]. The enzyme DES1–2 also plays a gatekeeper role in the production of ceramide from dhCer. In cellular experiments utilizing the DES1 inhibitor fenretinide, cells have a tendency to increase dhCer with significant reductions in apoptosis. Even when particular subsets of the pathway enzymes are targeted there is a compensatory increase in the other enzymes, producing biological effects that are specific to that enzyme subtype. For example, depletion of CerS2 leads to compensatory upregulation of CerS6 and therefore an increase in C16:0 ceramides.

Ceramides serve as both structural components of cell membranes and bioactive mediators in key cellular signaling pathways and homeostasis. In normal physiology, ceramides regulate a variety of essential processes including apoptosis, membrane integrity, stress adaptation, and insulin signaling modulation [36,37,38]. Ceramides contribute to apoptosis by serving as second messengers in intrinsic and extrinsic apoptotic pathways. They accumulate in response to cellular stress and can promote mitochondrial outer membrane permeabilization, facilitating cytochrome c release and activation of caspases [39]. Under physiological conditions, this apoptotic role is tightly regulated and contributes to normal tissue remodeling, immune surveillance, and turnover of damaged cells. Ceramides contribute to membrane integrity through their ability to induce coalescence of lipid rafts—rigid and ordered microdomains within the cell membrane that act as platforms for numerous cellular signaling processes [32,40]. This structural function supports efficient cellular communication and membrane dynamics during normal cellular functions.

Ceramides also act as critical mediators of the cellular stress response to oxidative stress, UV radiation, hypoxia, and inflammatory cytokines. Moderate ceramide accumulation in this context acts as an adaptive signal to pause cell proliferation, enhance repair, or trigger controlled apoptosis when damage is irreparable [41]. In terms of metabolic regulation, ceramides modulate insulin signaling by acting as negative regulators of the PKB (Akt/protein kinase B) pathway. Under normal metabolic states, ceramides help fine-tune insulin sensitivity and energy storage by providing feedback inhibition during nutrient surplus [42]. This negative feedback is enabled through promotion of Akt dephosphorylation via activation of PP2A (protein phosphatase 2A) or by preventing Akt translocation to the cell membrane via activation of PKCζ (protein kinase C zeta) [43]. In states of overt obesity, diabetes, and inflammation these mechanisms can become maladaptive, contributing to IR (insulin resistance). This balancing role played by ceramide underscores its dual nature as helpful in moderation but harmful in excess or chronic elevation. Ceramide levels can rise excessively due to aging, obesity, or systemic inflammation, contributing to CVDs such as HFpEF.

## 3. Current Paradigm of Ceramides in HFpEF Pathophysiology

Ceramide involvement in various disease states has been reviewed recently [44,45,46]. They have emerged as important bioactive lipids involved in HF and CVD and play crucial roles in systemic inflammation, metabolic syndrome, and diabetes [47,48,49], all of which are indicators for HFpEF diagnosis. In the failing myocardium of human and animal HFrEF models, accumulation of ceramide in both plasma and cardiac tissue is known to be involved in cardiac remodeling and dysfunction [50,51]. In HFpEF, biopsies of the myocardium are rare and scarce, and data on circulating ceramide levels in HFpEF are limited. Ceramide CVD risk is related to long-chain ceramides, especially C16:0, C18:0, and C24:0 [52,53]. For example, increased ceramides C16:0 and C18:0 in circulation were shown to be associated with death or heart failure admission in HFpEF patients in a subset of the TOPCAT trial (Treatment of Preserved Cardiac Function Heart Failure With an Aldosterone Antagonist) [54]. An important aspect of potential ceramide involvement in HFpEF pathophysiology is ceramide’s ability to impact cellular bioenergetics. Ceramide increases mitochondrial permeability, disrupting the electron transport chain, reducing adenosine triphosphate (ATP), and increasing reactive oxygen species (ROS). These lead to disruptions of mitochondrial bioenergetics, which impacts cells that have high energy demand such as cardiac myocytes [55,56,57]. This is important in HFpEF, where the cardiac energetic reserves are evidently reduced [58,59,60]. Recent findings further support the cardiac myocytes’ ability to produce and use ketones as energy substrates in the setting of HFpEF [61]. Furthermore, HFpEF therapies targeting cardiac energetics and metabolism such as semaglutide (glucagon-like peptide-1, GLP-1, agonist) and Dapagliflozin (Sodium glucose transport protein 2 inhibitor—SGLT2i) have been shown to reduce worsening HF [62,63]. HFpEF is increasingly being recognized as a cardiometabolic disorder, with several publications from our group and collaborators [58,61,64]. In addition to this, alterations in lipid metabolism due to metabolic disorders have recently being linked to HFpEF [1,65,66].

This link between metabolic disorder and HFpEF is of relevance to HFpEF pathophysiology, where the disease prevalence has increased in line with increasing obesity and diabetes as well as an aging population [67]. Both diabetes and obesity are categorized as metabolic disorders and are associated with abnormalities in lipidomics [68]. These contribute to IR and often lead to increased flux of fatty acids to other tissues including the heart, resulting in cardio-lipotoxicity fueled by the build-up of lipid species such as DAGs (diacylglycerols) and ceramide [69] and disruptions to other categories of lipids including sphingolipids. In addition, there are several unique sex-specific physiological and biological features, such as pregnancy and pregnancy-associated disorders, that can prime the heart for HF events, leading to HF phenotypes such as HFpEF as the female ages. Clinically, women with a history of multiple pregnancies have a more advanced HFpEF hemodynamic phenotype compared with women with no history of pregnancies, and those who develop HFpEF are more likely to have hypertensive disorders in pregnancy [16,70,71]. Example, increased plasma ceramide levels have been shown to be positively correlated with pre-eclampsia and the HELLP syndrome, particularly C16:0, C18:0, C18:1, C20:0, C22:0, and C24:1 [19,72]. Ceramide has also been proposed as a biomarker to track the progression of pregnancy-associated complications and guide clinical interventions [19]. Since HFpEF is a multifactorial disease, the proceeding sections assess ceramides’ effect in the setting of its risk factors.

## 4. Effect of HFpEF Risk Factors on Ceramide Biology

HFpEF is associated with a plethora of modifiable and non-modifiable risk factors. These include hypertension, diabetes, obesity, aging, and female sex. Each of these risk factors do have an impact on ceramide synthesis and signaling, which are summarized in Table 1.

### 4.1. Relationship of Estrogen Depletion and Ceramide

Increasing chronological age is a key non-modifiable risk factor for the development of HFpEF. In females this risk is compounded by a reduction in the sex-specific hormone estrogen due to ovarian aging or menopause. In the last two decades, several longitudinal studies have helped distinguish the effect of menopause and chronological aging effects on women’s cardiovascular health [13,14,73,74,75,76]. The strongest of this evidence was provided by the ongoing SWAN (Study of Women’s Health Across the Nation) heart study [14]. Data from these studies suggested that transitioning to menopause accelerates CVD risk factors such as shifts in body composition, increased blood pressure, worsening IR, and disruptions in lipid profile. In fact, ceramide levels within the cardiovascular system are known to increase with declining estrogen levels [17] and are comparably higher in older females (>63 years old) than males [77,78].

In menopausal women, the major form of E2 (17β-estradiol) is reduced by 80–90%, as E1 (estrone) becomes the main type of estrogen in circulation [79]. E2 is predominantly synthesized by the ovaries in pre-menopausal women. Other extra-gonadal sites, such as brain, heart, skin, bone, vascular endothelium, and aortic smooth muscle cells as well as adipose tissue, produce E2 in negligibly low quantities [80,81]. Reduction in E2 during and post-menopause significantly affects its role in counteracting inflammatory, apoptotic, endothelial dysfunction and cellular senescence signaling within the cardiovascular system [82,83,84]. E2 signaling can be both genomic and non-genomic through its receptors, ER*α*, ERβ (estrogen receptors alpha and beta), and GPER (G-protein-coupled estrogen receptor), which are encoded by *ESR1*, *ESR2,* and *GPER1* genes, respectively. E2 can modulate ceramide levels affecting energy balance and cellular processes. Evidence from E2-supplemented animal models of ovarian insufficiency have shown that E2 can reduce ceramide levels in the hypothalamus by reducing the rate-limiting enzymes SPT1 and SPT2 in the de novo synthesis pathway, conferring a protective role against obesity [85]. The main evidence for ceramide regulation by E2 comes from studies in human MCF-7 breast cancer cells [86,87]. In this cell line, E2 was able to increase cancer cell survival by stimulating the sphingolipid pathway enzyme, SphK, to convert ceramide to S1P. Ceramide primarily targets non-genomic E2 signaling pathways, particularly the pathways activated through cell membrane-associated estrogen receptors for cell survival such as PI3K/Akt (Phosphatidylinositol 3-kinase/Akt) and AMPK (AMP activated protein kinase) [88,89] and anti-inflammation signaling via inhibition of NF-κB (Nuclear Factor Kappa B) [90]. In the context of both chronological and ovarian aging, increased levels of ceramide impose opposite effects on these pathways, with increased cellular stress, apoptosis, and inflammatory signaling [91,92].

In women, estrogen has protective effects against ceramide accumulation, and its decline may exacerbate the effects of ceramide in the aging heart, contributing to higher prevalence of HFpEF in post-menopausal women [17]. Reduced estrogen could also indirectly increase cardiac stiffness and reduce autophagy, impairing cardiac function [93,94]. In a model of cardiac aging, E2 supplementation increased autophagy through activation of the key regulator of autophagy, Beclin 1, improving cardiac function [95]. In cardiac myocytes, states of ceramide overload increase autophagy/mitophagy, increasing mitochondrial stress and cardio-lipotoxicity [96]. An interesting aspect of myocardial stiffness and estrogen is related to the key cardiac structural protein titin and its phosphorylation by PKA (protein kinase A), PKG (reduced stiffness), and PKC (increased stiffness). Estrogen through ER*α* modulates titin’s subunit ratio by increasing cGMP-PKG (cyclic guanosine monophosphate–PKG) activation, leading to reduced stiffness [65]. These indicate the inverse (increased cardiac stiffness and auto/mitophagy) is true in states of estrogen depletion.

### 4.2. Contribution of Ceramide to Cell Senescence and Inflammaging

**Figure 2:**
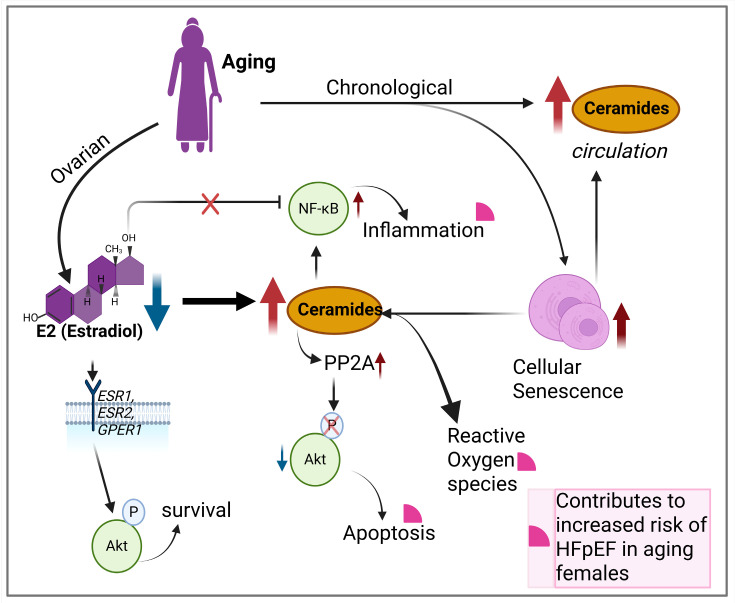
Ovarian and chronological aging result in elevated ceramide levels that contribute to increased inflammation, apoptosis, ROS, and increasing risk for HFpEF development. Blue arrow = reduction, red arrow = increase or accumulation, black arrows = forward effect and pink quarter circles = indicates contributors to HFpEF.

In addition to diminishing E2 levels, cell senescence plays a fundamental role in aging and inflammaging. Cellular senescence occurs in response to cellular stress or damage due to chronological aging. Ceramides also accumulate with age in circulation and multiple organs across various species [97]. Notably, the very long-chain C24:1 ceramide increases in serum extracellular vesicles with age in both humans and non-human primates, potentially contributing to cell senescence [98]. Ceramide levels in the female cardiovascular system have been shown to increase up to 4-fold in senescent cells [20]. Ceramide’s unique biophysical properties enable it to alter cellular membrane characteristics and trigger cell senescence events [99]. These events include DNA damage via mitochondrial ROS (reactive oxygen species) generation, cell cycle arrest, re-enforcement of senescence SASP (senescence-associated secretory phenotype), inflammatory signaling, and lysosomal stress. For example, ceramide localizes at biological membranes such as the outer mitochondrial membrane, where it self-assembles into stable, barrel-stave channels, allowing the selective release of pro-apoptotic proteins such as cytochrome c which lead to the activation of caspases [55]. Increased cytochrome c in the cytosol disrupts electron-transport-chain complex III and IV, causing electron leakage, generation of ROS, and diminished ATP (adenosine triphosphate) production [100,101]. Subsequently, increased ROS causes DNA damage and activation of DDR (DNA damage response), which is a hallmark of senescence [102]. Additionally, senescent cells exhibit SASP, a complex mixture of pro-inflammatory cytokines, chemokines, growth factors, and proteases, to reinforce senescence in both an autocrine and paracrine manner [103]. This affects the surrounding tissue microenvironment and contributes to aging and aging-associated disorders. Ceramide can contribute to this SASP-generated microenvironment by initiating, amplifying, or maintaining the activation of NF-κB [104,105] and the stimulation of MAPK (mitogen activated protein kinases) and JAK/STAT (Janus kinase/Signal transducer and activator of transcription) pathways [106,107]. Signaling through these pathways drive the transcription of inflammatory factors such as IL6 (interleukin 6), Il8, TNF-*α* (tumor necrosis factor alpha), and MMPs (matrix metalloproteinases). These can then contribute to inflammaging and exacerbate the risk for HFpEF development in the aging female (Figure 2) [108].

### 4.3. Ceramide’s Role in Hypertension

Hypertension is the most prevalent and well-established risk for HFpEF development. It accounts for nearly 80–90% of HFpEF cases [109]. Other risk factors can also induce hypertension through a complex interplay of factors that affect vascular homeostasis as the disease progresses. Vascular homeostasis encompasses a balance in redox signaling, anti- or pro-inflammatory signaling, mechano-sensing, and endothelial integrity [110]. Ceramide is able to contribute to or disrupt vascular homeostasis. However, there is greater emphasis on the link between elevated ceramide levels and its detrimental effects on vascular health through increased inflammation, oxidative stress, endothelial dysfunction, and neurohormonal dysregulation [111].

#### 4.3.1. Ceramide Promotes Vascular Oxidative Stress and Inflammation

Redox signaling in the vascular system is crucial to maintain a plethora of physiological processes including vasoconstriction and vasocontraction. The free-radical NO (nitric oxide) plays a key role in redox signaling. NO is made available from the conversion of l-arginine by eNOS (endothelial nitric oxide synthase), which is produced in the EC (endothelial cells) in response to shear stress or agonists such as acetylcholine [112]. NO then diffuses into the surrounding VSMC (vascular smooth muscle cell) where it activates sGC (soluble guanylate cyclase) to increase the production of cGMP. Elevated cGMP binds to PKG, causing a conformational change that activates PKG and leading to Ca^2+^ (calcium) reuptake into the sarcoplasmic reticulum which lowers intracellular Ca^2+^ and promotes vasodilation. Ceramide has been shown to trigger Ca^2+^ influx through recruitment of TRPC6 (transient receptor potential canonical 6) channels to the cell membrane [113]. This Ca^2+^ influx through TRPC6 channels activates the Rho/ROCK pathway, enhancing Ca^2+^ signaling and contraction and leading to vascular remodeling as observed in hypoxic pulmonary vasoconstriction studies [114,115,116]. Ceramide has also been shown to initiate the increased association of PP2A with eNOS in EC, leading to dephosphorylation of eNOS at ser1177 [117]. Dephosphorylation causes eNOS to uncouple, promoting the production of ROS such as superoxides instead of NO. Although short-term exposure of isolated human arteriole tissues to ceramide causes rapid switching of the mediators of flow-induced dilatation from NO to H_2_O_2_ (hydrogen peroxide) to preserve flow-induced dilatation [118], this can be detrimental in the long term [119]. Feng and colleagues [119] recently showed that ceramide 16:0 and ceramide 16:0/24:0 ratios were positively associated with cumulative coronary microvascular resistance in patients with coronary artery disease. This is not surprising since an increase in ROS in the vasculature eventually leads to oxidative stress and activation of redox sensitive transcription factors such as NF-κB, amplifying inflammatory gene expression and the activation of vasoactive proteins (ICAM-1, VCAM-1, E-selectin) within vessels. The knock-on effect of this is increased vascular stiffness and resistance, culminating in increased blood pressure or hypertension [112,120,121]. A clear connection between ceramides and oxidative stress in aging was made by a recent study comparing cardiac tissue from young and aged donor hearts with no known cardiac-related pathologies [122]. It is worth noting here that 7 out of the 13 aged donors were females aged between 50 and 65 years of age.

Interestingly, ceramide has been shown to activate the NLRP3 (NOD-like receptor protein 3) inflammasome in pulmonary microvascular EC [123]. NLRP3 is a key component of the innate immune system [124]. Ceramide activates NLRP3 via upregulation of the Txnip gene (thioredoxin-interacting protein), inducing the secretion of inflammatory cytokines [123]. HFpEF patients have been shown to have raised levels of circulating NLRP3, which is also linked to the pathogenesis of pregnancy complications including pre-eclampsia [125,126,127]. In women with pre-eclampsia, this inflammatory state can persist up to a median of 2 ½ years and contributes to the onset of HFpEF [16]. Additionally, enhanced systemic inflammation is thought of as a key player and a probable non-invasive diagnostic tool for HFpEF [128,129,130,131]. Unresolved ROS and systemic inflammation contribute to endothelial dysfunction.

#### 4.3.2. Ceramide and Endothelial Dysfunction

Endothelial dysfunction is a key disease mechanism for hypertension development and a feature of HFpEF, ultimately contributing to its progression [132]. ECs line blood and lymphatic vessels in the body and are an essential interface between blood and vessels in maintaining tissue homeostasis. Both a lack and accumulation of ceramide can cause endothelial dysfunction. A recent animal study showed that suppression of ceramide and S1P in the EC of high-fat-diet-fed mice underlies vascular and metabolic dysfunction [133]. Whether this depletion of ceramide in EC was a counter-regulatory mechanism of elevated ceramide levels in circulation or within the tissue microenvironment (EC isolated from heart and lung tissues) was not resolved in this study. This aspect is important since ECs have similar basic functions but show heterogeneity in terms of tissue microenvironment [134]. Human studies show that elevated plasma ceramides are associated with endothelial dysfunction in coronary and peripheral arteries [21,135]. Endothelial dysfunction is evident in aging and in women with HDP (hypertensive disorders of pregnancy) such as pre-eclampsia. In the latter, ceramide accumulation in the placenta leads to endothelial dysfunction, causing reduced tubular formation and increased trophoblast cell death [18]. Women with a history of HDP have a 2.09 times higher risk of hospitalization with HFpEF and an increased short- and long-term risk of incident HFpEF [16,136]. Diastolic dysfunction and LV remodeling are well-established features of pre-eclampsia.

Clinical studies have indicated specific ceramide long-chain species (d18:1/18:0, d18:1/24:1, ratio of (d18:1/18:0)/(d18:1/16:0)) as novel predictors of new onset hypertension [22] or as predictors of MACE (major adverse cardiac events) for hypertensive patients (CERT-HBP) identified to be at high CV risk [137]. In the myocardium, hypertension-induced ceramide accumulation could contribute to myocardial fibrosis and impaired diastolic relaxation, both of which are critical components of HFpEF. This is most likely through the activation of pathways such as MAPK and NF-κB [104,106], which promote inflammation and fibrosis in the heart, worsening the condition. Additionally, correlating the relationship between circulating ceramide and NLRP3 in pre-eclamptic or hypertensive female patients as a risk for HFpEF could provide diagnostic tools for HFpEF prevention in women.

### 4.4. Ceramide Biology in Metabolic Disorders

Metabolic disorders such as obesity and T2DM (type 2 diabetes mellitus) are strongly linked to the development and progression of HFpEF, with a higher prevalence in women than men [138,139]. Both disorders have prominent features of lipotoxicity and IR [140]. Lipotoxicity or the accumulation of lipids (including ceramides) within the circulation system due to increases in fat have deleterious effects on tissue glucose metabolism. Increased fat in the body occurs through the expansion (hypertrophy) or generation of new adipocytes (hyperplasia), which leads to an increase in abdominal, subcutaneous, and visceral adipocytes [141]. In post-menopausal women, abdominal fat distribution increases, possibly influenced by reduced E2 levels which have a negative correlation with ceramide levels [142,143]. Specific ceramide species such as d18:1/16:0, d18:1/18:0, and d18:1/24:1 accumulate in the visceral and subcutaneous tissue of diabetic or pre-diabetic females but not males [23]. Dysregulation of ceramide synthesis and degradation involving key enzymes in the sphingolipid synthesis pathways leads to ceramide accumulation and disrupts glucose metabolism in tissues such as the skeletal muscles, liver, and possibly the heart.

#### 4.4.1. Ceramides Influence on IR in Liver and Muscle

The skeletal muscle and liver play key roles in postprandial glucose handling [144,145]. Disruption of glucose metabolism in skeletal muscle and liver leading to the development of IR due to elevated ceramide levels is well characterized and reviewed [24]. IR develops prior to the onset of T2DM (type 2 diabetes mellitus) and can increase in severity as the disease progresses, with declining β-cell function and hyperglycemia. Recently, the co-existence of MASLD (metabolic-associated fatty liver disease) and HFpEF and their shared comorbidities, obesity and diabetes, have gained attention [146,147,148]. MASLD was found to be associated with a higher risk of developing HFpEF [148].

The type of ceramide species in circulation/tissue, which is linked to the fatty acyl-chain length preferences of the CerS1–6 enzymes, impacts insulin sensitivity. For example, animal models show that C16 and C18 ceramides modulate insulin sensitivity [149], while C24 and C24:1 have a protective role [150]. These studies targeted complete or partial depletion of CerS6 and CerS2 in the liver, respectively. The antagonistic role of ceramide C16 against insulin signaling and mitochondrial respiration has been attributed to its ability to regulate mitochondrial fission factor, which leads to the development of the insulin resistant phenotype [151]. Increased C16 ceramide levels in plasma have been linked to increased death and hospitalization in HFpEF patients [54]. Ceramide also interferes directly with insulin receptor signaling [152]. In normal physiology, insulin binds to insulin receptors, leading to the phosphorylation of insulin receptor substrates that in-turn activate the PI3K/Akt pathway. Ceramide targets AKT phosphorylation by increasing PP2A, reducing AKT phosphorylation [153] or directing PKCζ to caveolin-enriched microdomains to sequester AKT in a repressed state [154]. Ceramide’s blockade of AKT activity also impacts the plasma membrane translocation and fusion of GLUT4 (glucose transporter 4), thereby dysregulating insulin-dependent glycemic control [155].

In terms of skeletal muscles, two studies have shown evidence for muscular mitochondrial dysfunction in HFpEF patients aged >59 years old, and over 60% of patients were female [156,157]. Aging is known to shift mitochondrial dynamics towards fission and could further enhance IR [158]. Skeletal muscle dysfunction is also associated with exercise intolerance in HFpEF patients, apart from cardiac insufficiency [156,157,159,160]. Improved insulin sensitivity and glucose tolerance have been noted in skeletal muscles of mice lacking CerS1 [161]. Targeting genes of other enzymes in the de novo sphingolipid synthesis pathway, such as *Degs1* in other organs such as the liver, lowered skeletal muscle ceramide levels and improved insulin sensitivity [162]. This implies that circulating ceramides do have an impact on skeletal muscle glucose regulation. High concentrations of C16 and C18 ceramide produced by the ceramide synthase isoforms (CerS1, CerS5, and CerS6) play an important role in fat-induced skeletal muscle and hepatic IR [150,163]. Whether or not there is sexual dimorphism in the mechanisms of ceramide regulation within the liver and skeletal muscle in the absence of estrogen is currently unclear.

#### 4.4.2. Ceramides and IR in Obesity

Obesity impairs relaxation and filling of the heart, the key features of HFpEF [164]. Obesity also exacerbates vascular dysfunction and promotes pericardial fat accumulation, which enhances molecular and cellular inflammatory signals that drive HFpEF. An increase in fat depots increases recruitment of macrophages to the site, creating an inflammatory state characterized by increased cytokines such as Il-6, TNFα, and Il-1β [165,166,167]. Serum levels of TNF*α* often correlate with levels of ceramide or IR. In cultured cells TNF*α* stimulates ceramide biogenesis by inducing ceramide synthesis and sphingomyelin hydrolysis enzyme genes, which creates a vicious cycle of ceramide production and inflammatory signaling [168,169]. Ceramide-induced IR is partly attributed to the adipokine adiponectin and its receptors AdipoR1/AdipoR2 which improve insulin sensitivity by catabolism of tissue ceramide [170,171]. AdipoR1/AdipoR2 have intrinsic ceramidase activity and catalyze ceramide into S1P, improving insulin sensitivity and metabolic health. Adiponectin is encoded by the ADIPOQ gene and secreted in three known isoforms by adipocytes, with the high-molecular-weight (HWM) multimeric adiponectin being the most bioactive and negatively correlated to obesity [172]. This relationship has been observed in a study in post-menopausal women with BMI > 24 [173]. Furthermore, reduced adiponectin levels in pregnancy contribute to IR, as seen in gestational diabetes [174]. Increasing IR in HFpEF is also associated with worsened LV strain and MACE [175,176]. It is not clear if the changes during gestation, such as increased IR and reduced adiponectin, contribute to the reduced adiponectin and increased ceramide post-menopause, exacerbating obesity-related effects on the heart.

**Table 1:** Main triggers and mechanisms affecting ceramide biology in HFpEF risk factors.

Risk Factors	Main Triggers	Mechanisms	References
Aging + Female sex	Estrogen depletion	Increased ceramide due to reduced E2 effect on SPT1 and 2, reduced protection against obesity	[85]
		Increased inflammation, cellular stress, and apoptosis	[88,89,90]
		Increased cardiac stiffness through titin subunit modulation	[89]
	Cellular Senescence	4-fold elevation of ceramides in circulation	[20]
		Ceramide increases mitochondrial ROS generation and DNA damage	[177]
		Amplifying the inflammatory effects of senescence-associated secretory proteins (SASPs) through NF-κB MAPK and JAK/STAT pathways	[104,106,107,108]
Hypertension	Reduced NO availability	Ceramide triggers Ca^2+^ influx through TRPC6 channels	[113]
		Dephosphorylation of eNOS increasing ROS	[117]
	EC dysfunction	Activation of NLRP3 inflammasome	[123]
		Elevated ceramide associated with endothelial dysfunction in coronary and peripheral arteries	[135]
Metabolic Disorders	Insulin resistance +/− Obesity	Ceramide regulates mitochondrial fission factor	[151]
		Direct interference of insulin receptor signaling in liver and muscle through Akt modulation	[153,154]
		Reduced adiponectin levels due to obesity or pregnancy increases tissue ceramides	[170,171,174]

## 5. Ceramide Effects on Cardiac Metabolism

Metabolic dysfunction in HF is widely known. In HFpEF, this is an area of evolving interest. The current trajectory of research indicates HFpEF as a metabolic syndrome that is pronounced in females and has an interdependence of lipid use with cardiac hemodynamics [58], perhaps highlighting the potential mechanisms through which the SGLT2 inhibitors were improving cardiac function in HFpEF patients [178]. In the failing myocardium, ceramide levels are elevated, and this has been described as a contributor to clinical HF in the Framingham Heart Cohort [8,50]. Evidence from a recent in vitro model of ischemia/reperfusion injury in mice and hiPSC (human induced pluripotent stem cells) cardiomyocytes supports ceramide accumulation in myocytes when the enzyme CerS2 was over-expressed [179]. Ceramide’s effects on cardiac metabolism can be ascribed to its role in IR, inflammation, and arthrosclerosis. These effects can be conferred through ceramide’s downstream bioactive intermediate metabolites such as S1P and dhS1P (dihydrosphingosine 1 phosphate). We have reported hypertrophic and fibrotic effects of these metabolites on ventricular fibroblasts and myocytes isolated from neonatal rats that were comparable to angiotensin II [180,181]. However, in ischemia/reperfusion injury animal models (leads to HFrEF phenotype) these metabolites have a more protective effect [182,183], implying a disease-etiology-driven response. In fact, metabolic dysfunction in the heart is evidenced by the heart switching substrate type to maintain homeostasis in normal physiological and disease states [184]. Ceramide impacts mitochondrial function (as described earlier), which plays a vital role in this substrate switching response.

## 6. Current Recommended HFpEF Therapies and Their Effect on Ceramides

Several diabetes drugs have been trialed as management strategies for HFpEF, including the GIP (glucose-dependent insulinotropic polypeptide) and GLP1 agonists—semaglutide and tirzepatide—and SGLT inhibitors—dapagliflozin and empagliflozin. GLP-1 agonists, currently being trialed for HFpEF, have been found to be effective in reducing hospitalisations, including in those with obesity-induced IR [185,186]. Several lines of evidence show GLP-1 receptor agonists can inhibit ceramide generation, leading to reductions in serum levels of C16 and C24:1 ceramide species [187,188]. This reduction in ceramide may be a result of GLP-1R-enhanced metabolism of ceramide through alkaline ceramidase 2 (Acer2), and attenuation of TNFα induction by toll-like receptors, leading to a reduction in inflammation-induced ceramide accumulation or an increase in adiponectin and resulting in increased ceramide catabolism [187,189,190]. A clinical study reported reduced ceramide and its precursor metabolite, DhCer, in type 2 diabetes patients receiving liraglutide (GLP-1R agonist) and it was recommended as a CVD prevention therapy in a post hoc analysis of a randomized clinical trial showing reduced ceramides [191,192]. This implies that this therapy has either a direct or indirect effect on the de novo sphingolipid synthesis pathway. Despite this clinical evidence, there is a lack of mechanistic studies on the effects of current recommended therapies on ceramide biology in the context of HFpEF. This highlights a major need for more research in this area.

Interventions targeting ceramide metabolism show promise in improving health-span and potentially life-span [193]. For instance, inhibiting ceramide synthesis with myriocin enhances glucose homeostasis and grip strength in mice [97], and dietary interventions targeting older adults (55 to 80 years, >50% females) lead to a reduction in ceramide levels and CVD risk score [52]. It is understood that the prevalence of HFpEF increases in aging due to cellular aging, myocardial stiffness, and multiple comorbidities. At the cellular level, ceramides interfere with the function of EC, smooth muscle cells, and cardiomyocytes, leading to impairment of NO signaling and promotion of fibrosis which increases myocardial stiffness [194,195,196]. Ceramide is also known to activate stress kinases such as JNK (c-Jun N-terminal kinase) and p38 MAPK (mitogen-activated protein kinase) which increase the oxidative stress response in aging and can exacerbate myocardial stiffness [106,197,198], a hallmark of HFpEF. Therefore, interventions targeting ceramide could be beneficial for CVD health in the female aging population. However, there are limitations to targeting ceramides for therapy. The most obvious of these include ceramides’ complex biology and the heterogeneity of HFpEF pathophysiology, both of which have the possibility of introducing off-target effects and exacerbating the condition.

## 7. Pregnancy and Its Influence on Ceramide Biology

In addition to the risk factors mentioned above, recent data suggests a history of multiple pregnancies could be a risk for HFpEF development [70,199]. Ceramides play a significant role in pregnancy, with levels increasing towards the end of gestation in conditions such as pre-term labor and pre-eclampsia [25,26,200,201]. These unique temporal gestation patterns of ceramide levels highlight a potential unique role in gestation [202]. Ceramides also act as stress mediators during implantation, delivery, and lactation [19]. Elevated plasma ceramides during pregnancy are associated with IR and metabolic changes [26,203]. These changes may have implications for endothelial dysfunction, gestation diabetes mellitus, and fetal development [26]. Hormonal fluctuations in pregnancy are fundamental to successful pregnancy and birth of the child. Ceramides play a multifaceted role in metabolism and the signaling of steroid hormones. Ceramides can inhibit progesterone biosynthesis in rat luteal cells and granulosa cells, potentially contributing to luteal regression [204,205]. For example, in granulosa cells, ceramides mediate the inhibitory effects of IL-1β on progesterone production and enhance prostaglandin E2 biosynthesis. Ceramides are also involved in estrogen-modulated processes, as indicated earlier. What is yet to be researched is the genetic and epigenetic effects these changes may have and how they influence age-dependent disease development and progression as it relates to the pathogenesis of HFpEF in an aging female. This is especially important in terms of the effects of multiple pregnancies on heart health post-menopause, since a single pregnancy has tremendous cardiac and hemodynamic effects on the mother. Women with a history of multiple pregnancies (>3 pregnancies) tended to have an exaggerated hemodynamic phenotype than those who did not in female HFpEF patients [70]. Others have found multiparity to be associated with poorer cardiovascular health, even when adjusted for confounders such as job strain [206,207]. This association was later supported by a meta-analysis study showing multiparity has a dose-response relationship to CVD risk compared to nulliparity [208]. The analysis included 10 cohort studies involving over 3 million participants, with 150,512 incident cases of CVD. This is further supported by recent findings from analysis of HF risk within the UK Biobank showing multiparity to be associated with increased risk of HF [209]. This study emphasized the importance of female reproductive history in the assessment of HF risk. Additionally, we have published evidence of persistent transcriptomic changes in the myocardium of aged (24 month old) mice with a history of multiple pregnancies versus age-matched virgin mice [199]. Despite these recent efforts showing increasing risk of CVD with parity, there is a lack of comparable data in terms of differences in ceramide levels over the reproductive history between women with a history of multiparity and no pregnancy to clearly understand the potential contributions of ceramide to HFpEF in multiparous women. This lack of evidence supports the need to stratify studies well to capture such effects, if any. Such efforts would help determine whether measuring ceramide levels can enhance current HFpEF diagnostic methods for females.

## 8. Conclusions

As the evidence for ceramide in HFpEF slowly builds, its role in the pathophysiology of HFpEF, particularly in women, is important to decipher. However, when considering the HFpEF risk factor-based evidence currently available that show ceramide dysfunction, and involvement in molecular processes such as inflammation, metabolic dysfunction, and endothelial dysfunction influenced by declining levels of estrogen with age, it should be noted that ceramide has the potential to be a biomarker for early detection and risk assessment for HFpEF in women. This assessment is currently hampered by the lack of clinical and population studies targeting women at different life-stages and obstetric conditions. Future research into ceramide’s role in HFpEF should account for sex differences as well as stratifying for life-stages and obstetric factors to inform targeted therapies for women. These considerations should be incorporated even in basic mechanistic studies in cells and animals, where possible. This could lead to targeted strategies for early diagnosis or intervention, especially for women with a history of multiple pregnancies, pregnancy complications, or post-menopausal changes who have developed hypertension and–or obesity as they age.

## Data Availability

Data sharing is not applicable. This is only appropriate if no new data is generated or the article describes entirely theoretical research. No new data were created or analyzed in this study.
